# Towards climate neutrality for household energy consumption

**DOI:** 10.1093/nsr/nwac083

**Published:** 2022-04-30

**Authors:** Ortwin Renn

**Affiliations:** Institute for Advanced Sustainability Studies, Germany

The Paris Agreement, reinforced by the 2021 conference in Glasgow, obliges all nations that have signed the agreements to reduce the release of greenhouse gases to zero within the next few decades. China's central government has made two signature commitments on climate, pledging that the country's carbon dioxide emissions will peak before 2030, and the country will achieve carbon neutrality before 2060, when the amount of carbon emissions into the atmosphere will be offset by enhancing the absorbing capacity of national forests and other compensation methods. This implies that electricity, heat and all traffic vehicles will be fueled by renewable energy sources unless compensated by expanded sink capacity for CO_2_ [1].

The paper by Shen *et al.* [2] provides an excellent numerical review of household energy consumption in rural China, focusing primarily on cooking but also on heating systems. They concluded that the contributions to energy supply from coal and biomass fuels were still dominant in the rural household energy sector, while electricity comprised <20%, in energy units, in 2017. There was a clear increase in gas consumption by 204% compared to 2012. The paper points out, though, that replacing firewood and other organic material for cooking has been a major step forward towards cleaner indoor air, less overall pollution levels and healthy home environments. Notwithstanding that this progress towards more environmentally friendly and healthy cooking practices is necessary and long overdue, it provides only marginal improvements to the climate balance of cooking and heating systems in China.

How would carbon emissions stand, if the trend for using solid fuels, including coals, and in particular for cooking and home heating, is unbroken? First, one can observe, as the paper by Shen *et al.* [2] demonstrates, that major transitions have taken place in urban areas where electric heating systems, as well as gas boilers, have been penetrating the heat market and replacing less efficient coal or biomass cooking and heating devices. Second, the replacement of wood and biomass from non-sustainable sources has been propelled forward during the last two decades, which is a step towards sustainable practice even if natural gas is used as a substitute. Third, replacing natural gas with green gas (based on hydrogen generated from wind or sun power) and replacing coal-fired power stations with nuclear and renewable systems are promising pathways to transform the energy system, step by step. However, the present pace of introducing renewable energy into household consumption is insufficient for reaching the 2060 goal, especially under stacked energy use [3]. The transformation towards gas will continue, but mostly natural gas, not green gas. Heat pumps, another alternative to fossil fuels, are available but not very popular in China. They have great potential to replace more fossil-fuel-burning devices [4]. Changes in carbon emissions and household contributions to the total should be elucidated.

China is not alone on its way to reaching two major goals simultaneously: (i) to improve air quality and enhance environmental protection by modernizing cooking and heating systems and (ii) to replace fossil fuels with renewable energy sources. In most countries of the developing world, the quest for cleaner cooking and heating practices has led to intensive efforts to introduce improved technologies, home education programs and efficient services. Here, China can play a pioneering role for many Asian, African and Latin-American countries. The second program, the replacing of fossil fuels with renewable ones, however, has just been started and will need additional incentives and technological innovations in order to become the dominant form of energy generation in all countries. A strong commitment to invest in green gas production, as well as the use of renewable and nuclear energy for generating electricity (including heat pumps), are vital for achieving climate neutrality by 2060. A program to launch this transition is required now and not in the distant future if the goal is going to be attained.

***Conflict of interest statement***. None declared.
